# Regulation of HIV-Gag Expression and Targeting to the Endolysosomal/Secretory Pathway by the Luminal Domain of Lysosomal-Associated Membrane Protein (LAMP-1) Enhance Gag-Specific Immune Response

**DOI:** 10.1371/journal.pone.0099887

**Published:** 2014-06-16

**Authors:** Rodrigo Maciel da Costa Godinho, Flavio Lemos Matassoli, Carolina Gonçalves de Oliveira Lucas, Paula Ordonhez Rigato, Jorge Luiz Santos Gonçalves, Maria Notomi Sato, Milton Maciel, Ligia Maria Torres Peçanha, J. Thomas August, Ernesto Torres de Azevedo Marques, Luciana Barros de Arruda

**Affiliations:** 1 Departamento de Virologia, Instituto de Microbiologia Paulo de Góes, Universidade Federal do Rio de Janeiro, Rio de Janeiro, Brazil; 2 Laboratorio de Dermatologia e Imunodeficiencias, LIM-56, Departamento de Dermatologia, Faculdade de Medicina, Universidade de São Paulo, São Paulo, Brazil; 3 Departamento de Imunologia, Instituto de Microbiologia Paulo de Góes, Universidade Federal do Rio de Janeiro, Rio de Janeiro, Brazil; 4 Enteric Diseases Department, Infectious Diseases Directorate, Naval Medical Research Center, Silver Spring, Maryland, United States of America; 5 Department of Pharmacology and Molecular Sciences, The Johns Hopkins School of Medicine, Baltimore, Maryland, United States of America; 6 Department of Infectious Diseases and Microbiology, Center for Vaccine Research, Pittsburgh, Pennsylvania, United States of America; 7 Departamento de Virologia, Fiocruz – Pernambuco, Recife, Brazil; Johns Hopkins School of Public Health, United States of America

## Abstract

We have previously demonstrated that a DNA vaccine encoding HIV-p55*gag* in association with the lysosomal associated membrane protein-1 (LAMP-1) elicited a greater Gag-specific immune response, in comparison to a DNA encoding the native *gag. In vitro* studies have also demonstrated that LAMP/Gag was highly expressed and was present in MHCII containing compartments in transfected cells. In this study, the mechanisms involved in these processes and the relative contributions of the increased expression and altered traffic for the enhanced immune response were addressed. Cells transfected with plasmid DNA constructs containing p55*gag* attached to truncated sequences of LAMP-1 showed that the increased expression of *gag* mRNA required p55*gag* in frame with at least 741 bp of the LAMP-1 luminal domain. LAMP luminal domain also showed to be essential for Gag traffic through lysosomes and, in this case, the whole sequence was required. Further analysis of the trafficking pathway of the intact LAMP/Gag chimera demonstrated that it was secreted, at least in part, associated with exosome-like vesicles. Immunization of mice with LAMP/*gag* chimeric plasmids demonstrated that high expression level alone can induce a substantial transient antibody response, but targeting of the antigen to the endolysosomal/secretory pathways was required for establishment of cellular and memory response. The intact LAMP/*gag* construct induced polyfunctional CD4^+^ T cell response, which presence at the time of immunization was required for CD8^+^ T cell priming. LAMP-mediated targeting to endolysosomal/secretory pathway is an important new mechanistic element in LAMP-mediated enhanced immunity with applications to the development of novel anti-HIV vaccines and to general vaccinology field.

## Introduction

The magnitude and quality of the cellular and humoral immunological responses are crucial attributes for the development of an anti-HIV vaccine. Viral components can elicit a substantial immune response, as observed in long-term nonprogressors patients, and HIV-Gag structural protein seems to be particularly important in this context [1]–[3]. The presence of cellular immune responses directed towards this protein has been associated to the control of HIV infection both in the acute and asymptomatic stages and a strong anti-Gag CTL response is inversely correlated with the viral load in HIV-infected patients [3]–[6]. In addition, Gag is a well-conserved protein among different virus strains and subtypes, indicating that this protein is a target for the development of vaccines [7]–[9].

DNA plasmid based immunization has been shown to be a promising strategy in inducing immune response in different models [10]–[15]. The development of an anti-HIV DNA vaccine, however, is hampered by the fact that the expression of some viral proteins is dependent on viral regulatory elements. Specifically, the expression of HIV-Gag is critically dependent on Rev and Rev Responsive Elements (RREs) interactions for an efficient mRNA stability and translocation to the cytoplasm. Consequently, Gag protein expression is severely impaired in mammalian cells [16]. Several strategies have been used to overcome this Rev-dependence, like codon optimization and mutation of inhibitory sequences elements (INS) present along *gag-pol* sequence [17]–[19]. DNA immunization with these optimized sequences has been shown to elicit antibody and cytotoxic responses [20]–[22].

Stimulation of CD4^+^ helper T cells is essential for the induction of sustained CTL and antibody responses [23]. In this regard, an impaired ability to generate CD8^+^ T cells has been noticed in DNA vaccination systems, unless a CD4^+^ T cell response is also stimulated [24], [25]. Since Gag-specific CD4^+^ and CD8^+^ T cells proliferative responses are related to lower viral loads, an enhanced CD4^+^ T cell activation may be particularly critical for an effective HIV vaccine and for maintaining functional CD8^+^ T cell during chronic viral infection [5], [6], [26], [27].

The intracellular localization of an antigen can influence the magnitude and quality of humoral immune response and can also target the response to CD8^+^ or CD4^+^ T cells. In this regard, antigen targeting to different cellular processing compartments may improve its presentation by MHC I or MHC II molecules and enhance specific immune response [28]–[32]. In addition, the secretion of cellular proteins was reported to modulate the immunological responses. For instance, it was observed that a secreted form of HIV-Gag can induce a higher cellular response after DNA immunization than plasmids encoding a cytoplasmic form of this antigen [33]. Exosomes are endosome-derived vesicles commonly exploited by several cell types to secrete proteins. These vesicles are characterized by the presence of molecules related to the lysosomes, like CD81 tetraspanin, CD63, LAMP-1 and LAMP-2 [34]–[36]. Depending on the cell type, the exosomes may be originated from the invagination of the MIIC compartments and may also present proteins related to antigen presentation, such as MHC II, and co-stimulatory molecules, like CD86, what has been associated to increased vaccine efficiency [37], [38]. Indeed, exosomes derived from antigen presenting cells, such as B lymphocytes and dendritic cells are capable of antigen presentation and stimulation of T cells [39], [40]. Also, exosome derived from other cell types had been demonstrated to be directed to APCs, mediating antigen cross priming [41], [42].

We have previously shown that association of HIV-p55*gag* with mouse lysosomal associated protein-1 (LAMP-1), in a form of DNA vaccine chimera, promoted an enhanced Gag-specific immune response, in comparison to native *gag* DNA [28], [43], [44]. In vitro studies had also demonstrated that LAMP/Gag chimera was highly expressed and colocalized with MHCII in transfected cell lines [43]. However, the mechanisms regulating protein expression and intracellular targeting, as well as the relevance of each phenomenon in the enhanced immune response were not addressed yet. In the present study, we investigated the LAMP sequences necessary to modulate protein expression and intracellular targeting, and addressed which of these effects was associated to the increased immune response induced by LAMP/*gag* DNA vaccine. We observed that the association with LAMP-1 increases chimeric *gag* mRNA levels. The increased Gag expression in the LAMP/Gag context was dependent on LAMP-1 luminal domain and a minimum of 247aa of this region was necessary to increase antigen expression. The luminal domain also showed to be essential to target Gag to lysosomes and to induce Gag secretion. Increased expression was sufficient to induce a high acute antibody response in immunized mice. However, the enhanced CD4^+^ and CD8^+^ T cells activation, and prolonged antibody responses seemed to depend on Gag targeting to the endolysosomal/secretory pathway. We believe that the mechanistic study of the immune response induced by LAMP/*gag* is an essential step for the development of novel anti-HIV vaccines and may also contribute to the development of other LAMP-based vaccines.

## Materials and Methods

### Plasmids

Eukaryotic expression plasmids were constructed using nucleotides 1–1503 of the HIV-1 HXB2 p55*gag* gene (GenBank K03455) (HIV sequence Database, 1997, Los Alamos National Laboratory Theoretical Biology and Biophysics, Los Alamos, NM), inserted into pITR vector [45], which contains adeno-associated virus inverted terminal repeats (AAV-ITR) flanking the expression elements (CMV promoter and BGH polyadenylation signal). The LAMP/*gag* construct was made by inserting the p55*gag* (XhoI and EcoRI) sequence between the luminal domain (lum, between NheI and XhoI) and the transmembrane domain and cytoplasmic tail (TM-Cyt, between EcoRI and KpnI) of mouse LAMP-1 (GenBank J03881), as described previously [43]. The LAMP_lum_/*gag* construct was made by the same strategy, without the TM-Cyt insert.

The LAMP_REV-lum_/*gag* construct was made by replacing the luminal domain of LAMP-1 by its oriented reverse sequence. The LAMP-1 luminal domain in the reverse orientation was made by PCR, using the sequence 5′ccg.ctc.gag.atg.gcg.gcc.ccc.ggc.gcc.cgg.c 3′, (with the XhoI site) as the sense primer and the sequence 5′cta.gct.agc.cat.gtt.gtt.acc.atc.ctg.aac 3′, (with the NheI site) as the anti-sense primer. In this plasmid, a kozak sequence was added in the 5′ end of p55*gag* sequence. The plasmids containing the truncated LAMP-1 luminal domain were constructed by maintaining one third (372 bp; 124aa) or two thirds (741 bp; 247aa) from the 5′ end of the luminal domain. Therefore, the same sense primer used for the construction of LAMP/*gag* plasmid was used to make the truncated ones. The anti-sense primers used to make these plasmids were the following sequences: LAMP_TM-Cyt_/*gag* (24aa of LAMP lum): 5′ ccg.ctc.gag.agc.tga.ggc.gcc.atg.tgc 3′; LAMP_T1-lum_/*gag* (124aa of LAMP lum): 5′ccg.ctc.gag.att.ggg.aaa.atg.ttc.tgt.atc 3′; LAMP_T2-lum_/*gag* (247aa of LAMP lum): 5′ccg.ctc.gag.gaa.cgc.tct.ggt.cac.cgt.ctt 3′. All the plasmids were produced by transforming DH5α E. coli (Invitrogen, Carlsbad, CA) and purified with endotoxin-free columns (Qiagen Inc., Valencia, CA).

### Pulse and chase and immunoprecipitation

Cells from 293 cell line (HEK293, ATCC, Rockville, MD) were cultured in 6 well plates, at 5×10^5^ cells/well, in RPMI-1640 medium, containing 10% FCS, 2 mM L-glutamine and 100 U/ml of penicillin/streptomycin (Invitrogen). The cellswere transfected with pITR *gag* or pITR LAMP/*gag* (2 wells/plasmid), using Lipofectamine-2000 transfection reagent (Invitrogen), according to the manufacturers' protocol. Following 24 hours of culture, the medium was changed by a methionine-free RPMI medium (Invitrogen) and the cells were starved for 45 min/37°C. Then, 25 µCi of S^35^ labeled methionine (Amersham Pharmacia Biotech) were added to each well and the plates were incubated for 45 min/37°C. The cells were washed, the medium changed by a cold RPMI, and the cells and supernatants of these cultures were collected after 20 min, 1 hour and 6 hours. The cell samples were lysed with lysis buffer and both the cell lysates and supernatants were immunoprecipitated with anti-Gag antibody. Initially, the samples were pre-cleared by incubation with fixed SaC (Calbiochem-Novabiochem Corporation, San Diego, CA) for 1 hour, on ice, pelleted and incubated with normal goat serum (NGS) and SaC for 1 hour more, on ice. The pellets were incubated with mouse anti-Gag at 20 µg/ml in PBS, containing 1% BSA, overnight at 4°C and, then, incubated with 10 µg of purified goat anti-mouse IgG, on ice, for 1 h. The SaC was, then, added and the samples incubated for 20 min/ice, after what, the samples were washed twice with Pastan buffer (50 mM Tris, 5 mM EDTA, 100 mM NaCl, 2 M KCl, pH 7.5). The pellets were resuspended in TEN buffer (100 mM Tris, 5 mM EDTA, 150 mM NaCl, pH 8.0) with 1% NP-40, centrifuged and the obtained pellets were resuspended in SDS-PAGE sample buffer. Each sample was resolved in SDS-polyacrylamide gels and transferred to Immobilon P membranes (Millipore, Bedford, MA). A molecular weight marker was used as a standard (Amersham Pharmacia Biotech). The amount of radioactivity in the bands corresponding to native Gag or LAMP/Gag was measured in a phosphoimager.

### Analysis of *gag* mRNA by quantitative real time RT-PCR

HEK293 cells were transfected with the indicated plasmids. After 24 hours, mRNA was obtained either from total cell preparation or from isolated nuclear and cytoplasmic fractions using trizol reagent (Invitrogen), as indicated by the manufacturer. To isolate nuclear and cytoplasmic fractions, the cells were incubated for 10 minutes with lysis buffer (50 mM TrisHCl pH 8.0, 100 mM NaCl, 5 mM MgCl_2_, 0.5% vol/vol Nonidet p40) followed by centrifugation for 10 minutes at 10.000 RPM at 4°C. The pelleted fraction corresponded to the nucleus and the supernatant to the cytoplasm. The obtained mRNA was treated with DNAfree kit (Applied Biosystems, Carlsbad, CA, USA) to remove any plasmid contamination. The cDNA synthesis was conducted using the AMV first strand cDNA synthesis kit (Invitrogen), according to manufacturer's protocol. The cDNA from total cell lysate or nucleus and cytoplasm fractions were quantified for HIV-gag presence using the SYBR green method (Applied Biosystems), according to the manufacturer's instructions using the specific HIV-gag primers P24-7r (5′CCC.TGA.CAT.GCT.GTC.ATC.A3′) and P24inf (5′GTC.CAA.AAG.CGA.ACC.CAG.ATT.GTA.A 3′). For cycling and quantification a StepOne equipment and software (Applied Biosystems) were used.

### Analysis of protein expression by western blotting

HEK293 cells were transfected with the indicated plasmids, as described above. After 48 h of culture, the supernatant and cells were harvested, the cells were disrupted with lysis buffer (10 mM Tris-HCl (pH 7.5) with 150 mM NaCl, 1% sodium deoxycholate, 0.1% SDS, 1% Triton X-100 and premixed protease inhibitors (Complete, Roche Applied Science, Mannheim, Germany), for 15 min on ice, and cellular debris was removed by centrifugation. The amount of Gag protein in the cell lysate and supernatant fractions was analyzed by western blotting. Initially, the samples were normalized according to the total protein concentration, as determined by BCA (Pierce, Rockford, IL). They were then resolved on 10% polyacrylamide gels, transferred to Immobilon membranes (Millipore, Bedford, MA), and blocked with PBS containing 5% nonfat dried milk. Molecular weight markers (Amersham Pharmacia Biotech, Piscataway, NJ) were used as standards. After washing with PBS-0.05% Tween 20 (PBS-T), the blot was probed with mouse anti-Gag (kindly provided by Dr. James K. Hildreth, The Johns Hopkins School of Medicine, Baltimore, MD) at a 1∶50 dilution for 2 h, washed three times and then incubated with peroxidase-conjugated goat anti-mouse IgG antibody (Jackson ImmunoResearch Laboratories Inc., West Grove, PA) at a 1∶10,000 dilution for 1 h. The membranes were also probed with anti-β-actin antibody (Santa Cruz Biotechnology, Dallas, TX), followed by anti-mouse IgG, as a loading control. Super Signal West Pico Chemiluminescent Substrate (Thermo Scientific) was used for protein detection according to the manufacturer's instructions. The ratio of interest protein/constitutive protein was determined using ScionImage software.

### Exosome isolation

HEK293 cells were transfected with the indicated plasmids, as described above and, after 48 hours the culture medium was changed by serum-free Hybridoma-SFM (Invitrogen), containing 2 mM L-glutamine and 100 U/ml of penicillin/streptomycin. After 48 hours more, exosomes were isolated, as described elsewhere [46], [47]. Briefly, the cells were separated and the supernatant centrifuged for 10 min at 200 *g* (pellet P1). The supernatant was removed and centrifuged twice for 10 min at 500 *g* (the pellets were pooled and are referred to as P2). Supernatants were sequentially centrifuged at 2,000 *g* twice for 15 min (the pooled pellets are referred to as P3), once at 10,000 *g* for 30 min (pellet P4) and once at 70,000 *g* for 60 min (pellet P5), being P5 enriched in exosomes. The amount of Gag protein in P1–P5 samples was analyzed by western blot or p24-ELISA (see below). Western blot analyses were performed as described previously. The membranes were probed with anti-Gag, or anti-CD81 (1∶500; Santa Cruz Biotechnology), or anti-CD63 (1∶1000; Santa Cruz Biotechnology); followed by anti-mouse IgG (Jackson ImmunoResearch Laboratories) at a 1∶10,000 dilution for 1 h. For further purification of exosomes, P5 was resuspended in 5 ml of 2.5 M sucrose, 20 mM HEPES/NaOH, pH 7.2. A linear sucrose gradient (2.0–0.25 M sucrose, 20 mM HEPES/NaOH, pH 7.2) was layered on the top of the exosome suspension in a SW32Ti tube (Beckman Instruments, Inc.). Gradients were centrifuged for 15 h at 100,000 *g*, after which 2-ml fractions were collected from the bottom of the tube. To collect membranes from these fractions, they were diluted with 3 mL of PBS and centrifuged for 60 min at 200,000 *g*, using a Sw55Ti rotor (Beckman Instruments, Inc.). The fractions were washed once more and each one was analyzed by dot blot, since this technique is more sensitive than western blot and residual sugar did not interfere in the detection (see below).

### Detection of p24 by ELISA

HEK293 cells were transfected with the indicated plasmids for 48 h and the content of p24Gag in the cell lysates and supernatants was analyzed by ELISA. ELISA plates were coated overnight with anti-Gag M1 antibody (kindly given by Dr. James K. Hildreth, JHU), diluted in 50 mM Tris, pH 9.5, at 10 µg/ml. The plates were washed with PBS-T and blocked with PBS 3% BSA for 2 h, at 37°C. The samples and HIV p24 standard were diluted in RPMI, supplemented with 10% FCS and 1% Triton X-100, and incubated in the ELISA plates overnight, at 4°C. After several washes with PBS-T, the plates were incubated with biotinylated anti-p24Gag (kindly given by Dr. James Hildreth, JHU), diluted at 1∶4,000 in PBS with 5% normal goat serum, 1% BSA and 0.05% Tween 20, for 2 h at room temperature (RT). The plates were then incubated with HRP-streptavidin, for 30 min, at RT, washed and developed with TMB (BD PharMingen, San Diego, CA, EUA). The reaction was stopped with 1 M H_2_SO_4_ and read at 450 nm analyzed using an ELISA reader (BioRad Laboratories Inc., Hercules, CA, EUA).

### Confocal microscopy

For the analysis of the intracellular trafficking of the constructed chimeras, we used the MHCII-expressing DCEK.ICAM.Hi7 mouse cells [43], [48] (kindly given by Dr. Susan Swain, The Trudeau Institute, Saranac Lake, NY). These cells were maintained in RPMI-1640 medium, and were selected every other week by adding to the culture 6 µg/mL micophenolic acid; 250 µg/mL xantin; 15 µg/mL hipoxantin (Sigma, St. Louis, MO) and 800 µg/mL geneticin (Invitrogen). For immunofluorescence assay, the cells were plated in 6 well plates over poly-D-lysin pre-treated coverslips and were maintained overnight in RPMI medium, at 37°C/5% CO_2_. The cells were, then, transfected with the indicated plasmids, and, after 48 hours, the coverslips were transferred to a 24 well plate, washed with PBS, fixed with 2% paraformaldehyde and blocked with 4% normal goat serum and 0.1% saponin. To analyze Gag localization, the cells were stained with mouse anti-Gag antibody at 1∶50 dilution, washed with 0.1% saponin and stained with texas-red conjugated anti-mouse-IgG at 1 µg/mL (Jackson ImmunoResearch Laboratories Inc.). Endogenous LAMP molecules were detected by incubating the coverslips for 1 hour with rat anti-mouse LAMP-2 (ABL-93) supernatant medium, diluted 1∶50, and endogenous Golgi compartments were detected by staining with ABL-85 supernatant medium, diluted 1∶50. The coverslips were, then, incubated for 1 hour with FITC-conjugated anti-rat IgG at 1 µg/mL (Jackson ImmunoResearch Laboratories Inc.). The coverslips were washed with PBS and mounted onto glass slides, using ProLong antifade reagent (Molecular Probes, Eugene, Oregon). Confocal microscopy was performed using the Wallac confocal Laser Scanning Microscope and the images were captured individually and digitally coloured by using Photoshop 5.0 (Adobe, San Jose).

### Mice CD4^+^ T cell depletion

Female BALB/c mice, 6–8weeks age, were obtained from the mice facility of the Instituto de Microbiologia, Universidade Federal do Rio de Janeiro (IMPPG, UFRJ), Brazil. The animals were bred and housed according to institutional policies for animal care and usage and the protocol was approved by The Ethics Committee of Animal Care and Use (Comite de Etica no Uso de Animais-CEUA) from Centro de Ciencias da Saude, UFRJ (Permit Number: IMPPG 025). The mice (4 mice/group) were treated with purified rat IgG against mice CD4 molecule obtained from GK1.5 hybridoma (ATCC TIB-207; kindly provided by Dr. José Mauro Peralta, Universidade Federal do Rio de Janeiro, Brazil). Each animal received an intraperitoneal injection of 100 µg/100 µL/mouse for 3 days followed by a 4 days' rest. The injections continued along the whole experiment with an interval of 4 days between treatments. The efficacy of this procedure was evaluated by flow cytometry achieving 70–85% T CD4^+^ depletion (data not shown).

### Mice immunization

Female BALB/c mice, 6–8 weeks old, were immunized twice, i.d., with the indicated plasmids at 50 µg/50 µL/mouse, at a 3-4 weeks interval.

### Antibody response

Mice sera were obtained from the tail vein before the first immunization (pre-bleed) and at different time points after the second immunization, and the individual serum IgG levels were measured by ELISA. Briefly, ELISA plates were coated with 50 µL of HIV_IIIB_ lysate at 5 µg/ml (ABI, Rockville, MD) and incubated at 4°C, overnight. The plates were blocked with PBS containing 10% FCS for 2 hours/37°C, washed with PBS-T and the serum samples were added, in serial dilutions, and incubated at 4°C/overnight. The plates were, then, incubated with HRP-conjugated anti-mouse IgG (1∶5,000; Jackson ImmunoResearch Laboratories Inc.) for 2 hours/37°C, washed and developed using TMB substrate (Pharmingen, San Diego, CA). After 30 minutes, the reaction was stopped with 1 M H_2_SO_4_ and read at 450 nm using an ELISA reader (BioRad Laboratories Inc.).

### T lymphocyte activation

#### ELISPOT assay

The activation of CD4^+^ and CD8^+^ T lymphocytes was analyzed by ELISPOT assays, using the IFN-γ ELISPOT set from BD-Biosciences Pharmingen (San Diego, CA), according to manufacturer's protocol. Initially, ELISPOT plates were coated with anti-IFN-γ antibody at 5 µg/mL and incubated at 4°C/overnight. The plates were blocked with RPMI 1640, containing 10% FCS, for 2 hours at RT and then, total splenocytes (10^6^ cells/well), obtained from each immunized mouse, were cultured in the presence of culture medium (RPMI 1640 medium supplemented with 5% FCS, 100 units/mL penicillin/streptomycin, 2 mM L-glutamine, 50 µM 2-mercaptoethanol and 1 M HEPES buffer) or recombinant baculovirus HIV_SF2_p55 Gag (5 µg/mL; NIH AIDS Research and Reference Reagent Program), to analyze the CD4^+^ response; or with the MHC I restricted Gag epitope AMQMLKETI_65-73_ (10 µg/mL), to analyze the CD8^+^ response, as indicated in the results. After 24 hours of culture, the plates were washed and incubated with biotinylated anti-IFN-γ antibody for 2 hours at room temperature, followed by incubation with HRP-conjugated avidin, for 1 hour/RT. The reaction was developed with AEC substrate (Calbiochem-Novabiochem Corporation, San Diego, CA). Analysis of the IFN-γ levels was performed using the Immunospot Analyzer software (BD Biosciences, San Diego, CA). The data indicate the average number of spot forming cells (SFC) obtained from individual mice.

#### Analysis of cytokine production by ELISA

Splenocytes (10×10^6^ cells/mL) were cultured in triplicate in a 96-well plate in the presence of recombinant baculovirus HIV_SF2_p55 Gag, or 15-mers spanning the whole Gag protein, or T CD4^+^-restricted Gag peptide pools, or T CD8^+^-restricted Gag peptide pools [28]. All peptide pools were at the same concentration (5 µg/mL; NIH AIDS Research and Reference Reagent Program). As negative and positive controls, cells were incubated with culture medium alone or concanavalin A (ConA) (BD Biosciences), respectively. Culture supernatants were harvested after 72 h for the quantitation of secreted TNF-α or IFN-γ using an OPTEIA ELISA kit (BD Biosciences).

#### Analysis of CCR7 expression and intracellular cytokine staining by flow cytometry

Splenocytes harvested from the immunized mice were incubated with the described Gag peptides, in the presence of brefeldin A (eBiosciences Inc, San Diego, CA) for 10 h, at 37°C. After incubation, cells were washed twice with FACS buffer (HBSS, supplemented 2% FCS, 1 mM Hepes, 0.1% NaN_3_), and nonspecific binding was blocked by incubating cells with anti-FcγR antibody (BD Pharmingen) at 10 µg/mL for 15 min at 4°C. The splenocytes (1×10^6^ cells/well) were stained in duplicate with PerCP-conjugated rat anti-mouse CD4^+^ antibody, and/or PE-conjugated anti CCR7 (BD Pharmingen) at a dilution of 1∶100 for 30 min at 4°C. The cells were washed twice with FACS buffer and resuspended in 200 µL of Cytofix/Cytoperm solution at 4°C for 20 min. Cells were then washed twice with Perm/Wash solution and stained with APC-conjugated anti-IFN-γ, or APC-conjugated anti-IL-2 and PE-conjugated anti-TNF-α antibodies (BD Biosciences) diluted 1∶100. Events acquisition was performed with a FACScalibur flow cytometer instrument and data was analyzed with CellQuest software (BD Biosciences). A minimum of 50,000 events were analyzed.

### Statistical analysis

Statistical analysis of the results was based on unpaired t-tests and chi-squared independence tests. p values <0.05 were considered statistically significant.

## Results

### Association of HIV-Gag with LAMP-1 increases *gag* mRNA transcription

**Figure 1:**
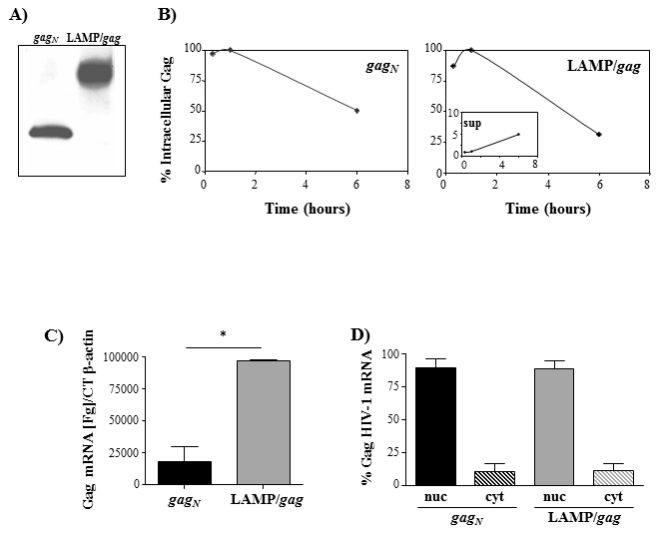
Association of HIV-1 gag with LAMP-1 does not protect Gag protein from degradation, but increases Gag mRNA production. A) HEK 293 cells were transfected with native *gag* (*gag_N_*) or LAMP/*gag*. After 48 hours, the expression of Gag protein was analyzed by western blotting, by staining with mouse anti-Gag antibody, followed by HRP-conjugated anti-mouse IgG. B) HEK 293 cells were transfected with *gag_N_* or LAMP/*gag*. After 24 hours, the cells were pulsed with S^35^ and, then, chased for 20 min, 1 h and 6 h. The cells and supernatants were collected and immunoprecipitated with anti-Gag antibody. The protein content in each sample was analyzed in a phosphoimager and the curves represent the proportion of protein observed at each time point in relation to the maximum value (100%); the insert indicates the values obtained in the supernatant (sup) of LAMP/*gag* transfected cultures. C and D) HEK 293 cells were transfected with the indicated plasmids and, after 48 h, total RNA was obtained from either whole cells (C) or from isolated nuclear (nuc) and cytoplasmic (cyt) fractions (D). After reverse transcription using oligodT primers, the amount of *gag* cDNA was evaluated by qPCR. Gag concentration were normalized using the β-actin CT number. Data is representative of three independent experiments. * p<0.05.

We have previously demonstrated that a DNA plasmid construct containing the sequence of HIV-p55*gag* inserted between the luminal and the transmembrane and cytoplasmic tail of LAMP-1 was highly expressed after transfection of different cell lines [28], [43]. In order to verify whether the increased Gag expression was related to a higher stability of LAMP/Gag protein chimera, in comparison to native Gag, we performed pulse and chase experiments and determined the degradation rate of each protein. HEK293 cells were transfected with either native *gag* (*gag_N_*) or LAMP/*gag* DNA plasmids. After 24 hours, the cells were pulsed with S^35^ and, then, chased for 20 minutes, 1 hour or 6 hours in cold medium, and the samples were immunoprecipitated with anti-Gag antibody. We observed that, while the amount of protein produced following transfection with the native *gag* DNA was much lower than the one produced by LAMP/*gag* [28], [43] (Figure 1A), their degradation curves were very similar (Figure 1B). The degradation rate of LAMP/Gag chimera was slightly higher, but this could be associated to the lysosomal targeting and secretion of this protein, since LAMP/Gag was also observed in the supernatant of transfected cells, whereas there was no appreciable amount of native Gag in the supernatant (Figure 1B, insert).

We then investigated if the modulation of Gag expression by LAMP would be correlated either to mRNA transcription or translocation to the cytoplasm. HEK293 cells were transfected with *gag_N_* or LAMP/*gag* plasmids and the mRNA was extracted from the whole cell lysate, or from isolated nuclear or cytoplasmic fractions. HIV-*gag* mRNA was then quantified. We observed a significant difference in the total mRNA concentration between *gag* and LAMP/*gag* (Figure 1C), but the distribution between the nucleus and the cytoplasm was very similar for both chimeric genes (Figure 1D), indicating that there was no difference in the translocation rate between these mRNAs. These results suggest that the presence of LAMP signals in the chimeric construct increased mRNA transcription or stability, resulting in higher steady state levels of LAMP/*gag* mRNA.

### The increased LAMP/Gag expression is mediated by LAMP-1 luminal domain

**Figure 2:**
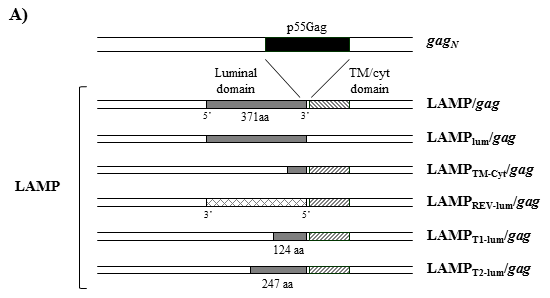
Schematic representation of the constructed plasmids containing different domains of LAMP-1 associated to p55*gag*. p55*gag* (black rectangles) sequence was inserted between the intact or truncated luminal domain of LAMP (gray rectangle) and the transmembrane and cytoplasmic tail of LAMP (TM/Cyt; striped rectangles).

**Figure 3:**
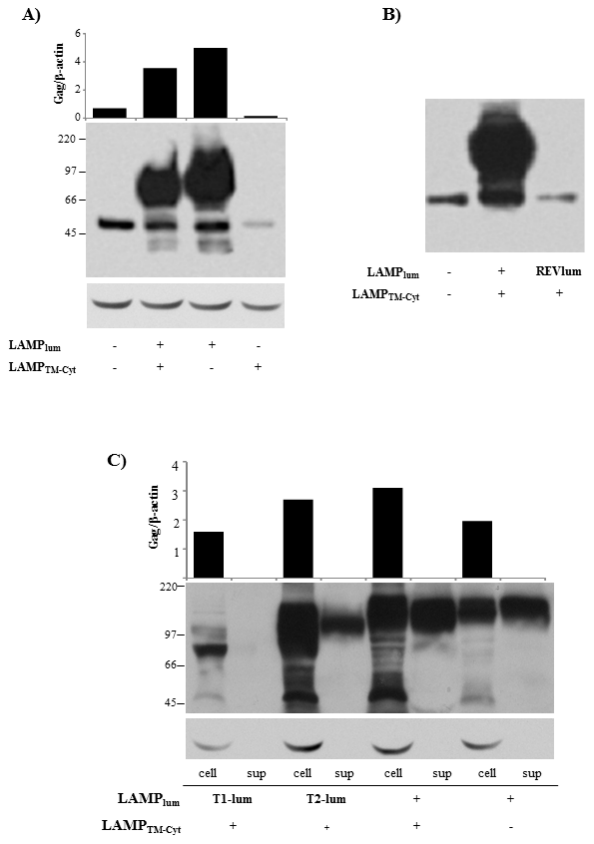
LAMP-mediated increased Gag expression is dependent on LAMP luminal domain. **A**–**B**) HEK293 cells were transfected with the plasmids represented in Figure 2. After 48 h, the amount of Gag protein was analyzed by western blotting, by staining with mouse anti-Gag antibody, followed by HRP-conjugated anti-mouse IgG. The membranes were also probed with β-actin, as a loading control. Bars indicate the ration between Gag expression and b-actin, as measured with ImageJ software. **C**) HEK293 cells were transfected with the indicated plasmids and, 48 h later, the amount of Gag protein in the cell lysates (cell) and culture supernatants (sup) were analyzed as in (**A**). Data is representative of four independent experiments.

We analyzed the role of the different LAMP-1 domains in the regulation of Gag expression. Several DNA plasmids were constructed by deleting either the transmembrane-cytoplasmic tail (TM-Cyt) (plasmid LAMP_lum_/*gag*), or the luminal domain of LAMP in the LAMP/*gag* chimera (plasmid LAMP_TM-Cyt_/*gag*) (Figure 2). In the latter construct (LAMP_TM-Cyt_/*gag*), we maintained 24aa of the 5′ terminal of LAMP, correspondent to ER signal sequence. HEK293 cells were transfected with these plasmids and Gag expression was analyzed by western blot (Figure 3). The result presented in figure 3A confirms our previous observation of an increased LAMP/Gag expression in comparison to native Gag, and demonstrated that the deletion of LAMP cytoplasmic domain did not affect Gag expression. In contrast, deletion of the luminal domain led to a decreased Gag expression to the level obtained with native Gag. Additionally, cell transfection with a chimeric plasmid containing the LAMP luminal domain in a reverse orientation at the 5′end of the start site of the *gag* gene also showed a decreased Gag expression, comparable to native Gag (Figure 3B).

To identify the minimum sequence of LAMP luminal domain necessary to increase Gag expression, we generated two other truncated LAMP/Gag chimeras, with deleted sequences from the 3′ end of the LAMP luminal domain. One of the constructs, LAMP_T1-lum_/*gag,* contained one third (372 bp–124aa) of the luminal domain; and the other, LAMP_T2-lum_/*gag*, contained two thirds (741 bp–247aa) of the luminal domain (Figure 2). HEK293 cells were transfected with these chimeras and their expression in the cell lysate or culture supernatant were compared with the ones observed with the complete LAMP/*gag* construct and with the construct containing only the whole luminal domain (LAMP_lum_/*gag*). The amount of protein detected was proportional to the length of LAMP luminal domain and a minimum of 247aa of the luminal sequence was necessary to achieve an optimal expression level (Figure 3C). In addition, cell transfection with the intact LAMP/*gag* or with LAMP_lum_/*gag* induced the secretion of high levels of Gag protein and, similarly, a minimum of 247aa was required to induce Gag secretion, although at a lower level (Figure 3C).

### An intact LAMP luminal domain is necessary to target Gag to lysosomes and exosome secretory pathway

**Figure 4:**
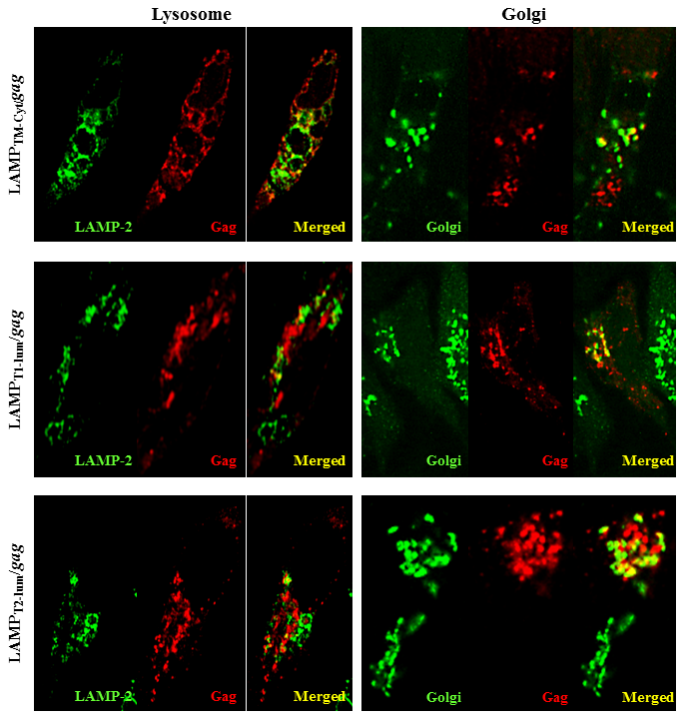
Truncated LAMP/*Gag* chimeras poorly colocalize with endogenous LAMP. Mouse DCEK cells were transfected with the indicated plasmids. After 48-Gag antibody, followed by incubation with texas-red anti-mouse IgG. Colocalization with Golgi compartments was analyzed by staining with a rat anti-mouse golgi gp125, followed by incubation with a FITC-conjugated goat-anti-rat IgG. Colocalization with endogenous LAMP was analyzed by staining with a rat anti-mouse LAMP-2, followed by incubation with a FITC-conjugated goat-anti-rat IgG. Data is representative of four independent experiments.

We analyzed how much of the LAMP luminal domain was required to promote Gag-targeting to the lysosomal compartments. Mouse DCEK cells were transfected with the truncated LAMP/Gag chimeras and their localization in lysosomes or Golgi complex were analyzed by confocal microscopy, using anti-LAMP-2 and anti-gp125 (ABL85 hybridoma) antibodies, respectively. We observed that the truncated LAMP/Gag chimeras were scarcely present at lysosomes in transfected cells, but they all showed strong colocalization with Golgi apparatus (Figure 4), suggesting that without essential sequences present in luminal domain, the chimeras are retained at these compartments and are not able to traffic through lysosomal/secretory pathway.

**Figure 5:**
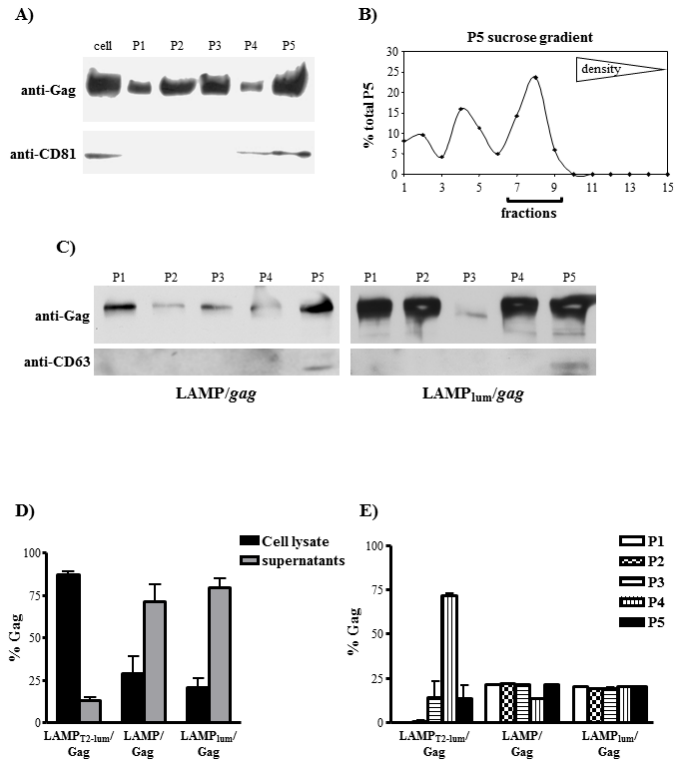
LAMP luminal domain induces LAMP/*Gag* secretion through exosomes. **A**) HEK293 cells were transfected with LAMP/*gag* and, after 48 hours, the supernatants were harvested and subjected to differential centrifugations as described in Material and Methods. Cell lysates (cell), the intermediate pellets obtained after each centrifugation step (P1–P4), and the exosome fraction (P5) were obtained and Gag expression was analyzed by western blotting. The membranes were also probed with anti-CD81, as an exosome marker. **B**) P5 fraction obtained as in (**A**) was brought to 2.5 M sucrose, overlaid with a continuous sucrose gradient and subjected to equilibrium centrifugation. Fifteen fractions were collected and subjected to dot blot analysis using anti-Gag antibody. Densitometry analysis was performed using phosphoimager software and the results indicate the percentage of each dot intensity in relation to the sum of all dots. Membrane-associated fractions are indicated with a line in the bottom. **C**) HEK293 cells were transfected with LAMP/*gag* or HEK293 cells were transfected with LAMP_lum_/*gag*. After 48 hours, the supernatants were harvested and subjected to differential centrifugations as described. P1–P5 fractions were analyzed by western blotting, using anti-Gag or anti-CD63 antibodies **D**–**E**) HEK293 cells were transfected with LAMP_T2-lum_/*gag*, LAMP/*gag* or LAMP_lum_/*gag*. After 48 h, cells and supernatants were harvested, fractionated as in (**A**), and the concentration of Gag was measured by p24-ELISA. The proportion of Gag protein in the cell lysates and total supernatant fraction is demonstrated in (**D**); the percentage of Gag in each supernatant fraction in relation to total supernatant is demonstrated in (**E**). Data is representative of three independent experiments.

Since previous reports demonstrated the presence of LAMP in exosomes in some cell types [34], [49], we investigated whether LAMP/Gag was being secreted in these vesicles. The supernatant of LAMP/Gag-transfected cells was submitted to serial centrifugations up to 70,000 g (pellet 5-P5), where the exosomes are usually enriched. Western blot analysis demonstrated that Gag was present in all supernatant fractions, including P5, where the CD81 tetraspanin was also enriched, strongly suggesting that this is actually related to exosomes (Figure 5A). Still, since Gag protein can form aggregates, and these could be pelleted in the same fraction as the exosomes, we further purified the P5 fraction in sucrose gradients and observed the presence of Gag in the fractions related to membrane conjugates, confirming that LAMP/Gag is partially secreted in exosomes (Figure 5B). To further confirm the importance of the LAMP-1 luminal domain in promoting Gag traffic through lysosomes/exosomes vesicles, we also investigated fractionated supernatants form HEK293 cells transfected with LAMP_lum_/*gag*, which contain only the luminal domain of LAMP. Similar to complete LAMP/gag, LAMP_lum_/Gag chimera was strongly expressed in all supernatant fractions, including P5 (Figure 5C). The membranes were also probed with anti-CD63, another exosome marker, which was also enriched in P5 fraction, corroborating the previous data. These data suggest that the whole LAMP luminal is necessary and maybe sufficient to target Gag to the exosome secretory pathway.

We could not detect the LAMP_T2-lum_/Gag chimera in the fractionated supernatants by western blot (data not shown). Therefore, we also analyzed the Gag protein amount in the cellular lysate and in all supernatant fractions isolated from LAMP_T2-lum_/g*ag*, LAMP/*gag* or LAMP_lum_/*gag* transfected cells by p24 ELISA. Initially, we determined the proportion of Gag in the total supernatant in comparison with cell lysates. After cell transfection with LAMP_T2-lum_/Gag, 15% of total Gag was present in the supernatants and around 85% was present in the cell lysates. In contrast, samples obtained from LAMP/*gag* and LAMP_lum_/*gag*-transfected cells showed around 70 and 75% of Gag in the supernatants, respectively (Figure 5D). After fractionating the supernatants, we observed that LAMP_T2-lum_/*gag* were highly enriched in the P4 fraction, differently from LAMP/*gag* or LAMP_lum_/*gag*, that were also enriched in P5 (Figure 5E).

### LAMP luminal domain-mediated high expression and targeting to lysosomal/secretory pathway promote an enhanced anti-Gag immune response

**Figure 6:**
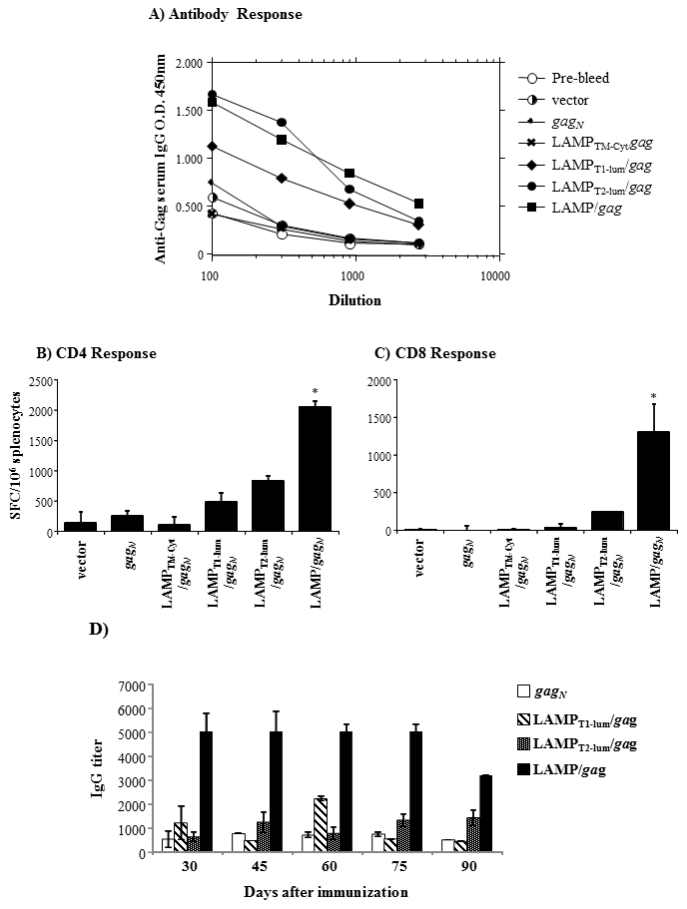
High protein expression and targeting to secretory pathway induced by LAMP luminal domain potentiate the immune response to HIV-Gag. Balb/c mice were immunized twice with the indicated plasmids, i.d, at 50 µg/mouse. A) The serum of each mouse was collected before the immunization (pre-bleed) and 10 days after the second immunization and the amount of anti-HIV IgG was measured by ELISA. The curves indicate the average O.D. levels obtained at different serum dilutions. B–C) Total splenocytes obtained from individual mice were cultured with p55Gag protein (B), or with the MHC I restricted Gag epitope AMQMLKETI65-73 (C) and IFN-γ production was analyzed by ELISPOT assay. The bars indicate the average of SFC/10^6^ cells, subtracting the values obtained with medium only. D) Mouse serum of individual mice was collected at the indicated time points after immunization with the indicated plasmids. The amount of anti-HIV IgG was measured by ELISA as in (A). The bars indicate the dilution point relative to 50% of the maximum O.D. (IgG titer). The data are representative of three independent experiments. * p<0.05.

We verified here that 247aa of the luminal sequence was necessary to promote high levels of Gag expression but only the complete and intact luminal domain was able to induce Gag-targeting to the endolysosomal/secretory pathway. In order to determine the effects of protein expression and cellular traffic on immune responses, mice were immunized with the native *gag* and with the different LAMP/*gag* constructs, and both the antibody and the cellular responses were analyzed (Figure 6). The amount of anti-HIV IgG was measured ten days after two DNA immunizations, and we observed that the construct containing 247aa of the luminal domain (LAMP_T2-lum_/*gag*) induced an antibody response level similar to the intact LAMP/*gag* (Figure 6A). In contrast, both the CD4 and, mostly, the CD8 T cell response, were remarkably impaired in the mice immunized with any of the truncated LAMP/*gag* constructs, in comparison to the DNA encoding the intact LAMP/*gag* (Figures 6B and 6C). These data suggest that high protein expression is sufficient to elicit a significant antibody response, but not cellular activation, which seems to depend on intracellular traffic. Interestingly, immunization of with LAMP_lum_/*gag* also increased production of IFN-γ by CD4^+^ and CD8^+^ T cells, as measured by ELISA and flow cytometry. The T cell response observed in LAMP_lum_/*gag* immunized mice were similar than the one induced by complete LAMP/*gag* plasmid, which was much higher than the response induced by native *gag* immunization (Figure S1A and B). These data indicate that, in the system presented here, association of Gag antigen with LAMP-1 luminal domain was sufficient to elicit a potent Gag-specific T and B cell-mediated immune response.

Since T cell, particularly CD4^+^ T cell priming, is important to induce and maintain memory response in general, we questioned whether the increased antibody production induced by truncated LAMP_T2-lum_/Gag construct would be sustained for longer periods of time.

Thirty days after the second immunization, the titer of anti-HIV serum IgG antibodies was similar between LAMP_T1-lum_/*gag*, LAMP_T2-lum_/*gag* and native *gag* immunized mice, and none of them were comparable to the ones induced by intact LAMP/*gag* construct. Consistent with our previous data, antigen-specific IgG antibody response induced by the LAMP/*gag* chimera was sustained at titers greater than 1∶3,000 for at least three months after the immunization (Figure 6D). Taken together, these results indicate that a combination of high expression and targeting to secretory cellular pathway promote more complete and long lasting responses.

### Immunization with LAMP/*gag* induces polyfunctional T cell response

**Figure 7:**
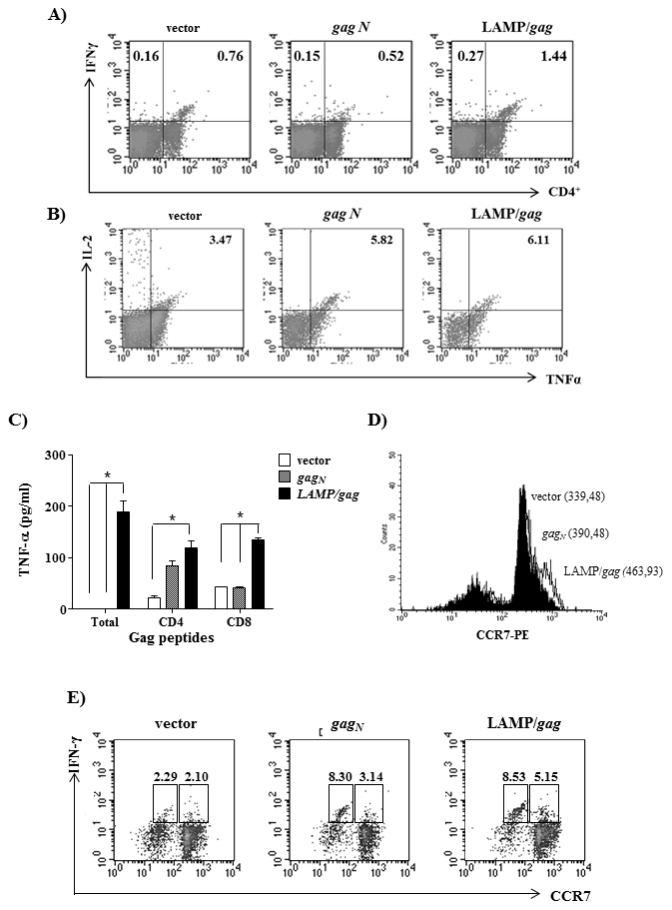
LAMP/*gag* immunization induces polyfunctional and memory CD4^+^ T cells. Balb/c mice were immunized twice with the indicated plasmids. Fifteen days after the second immunization, the splenocytes were cultured with CD4- or CD8-restricted Gag peptides and the phenotype and cytokine production were analyzed. **A**–**B**) The cells were stained with PercP-anti-CD4 and APC-anti-IFN-γ, or with PercP-anti-CD4, APC-anti-IL-2 and PE-anti-TNF-α, and analyzed by FACS. Dot blots indicate CD4 and IFN-γ staining (**A**) or TNF-α and IL-2 staining among CD4^+^ cells (**B). C**) Culture supernatants were collected and the amount of TNF-α was analyzed by ELISA. **D**–**E**) Cells were stained with PercP-anti-CD4, PE-anti-CCR7 and APC-anti- IFN-γ. CD4^+^ cells were gated and the percentage and/or mean fluorescence intensity (MFI) of CD4^+^CCR7^+^IFN-γ^+^ cells were analyzed by FACS. **D**) Histograms indicate CCR7 expression among CD4^+^ cells. pITR vectors are in black; *gag_N_* are in dashed line and LAMP/*gag* are in line histogram. The numbers indicated MFI values. **E**) Dot blots indicate the percentage of CCR7 and IFN-γ expression among CD4^+^ cells. Numbers indicate the percentage of IFN-γ^+^ cells among CCR7^hi^ and CCR7^l^°CD4^+^ gated cells. The data are representative of three independent experiments. * p<0,05.

An efficient vaccine response against HIV infection requires the activation of polyfunctional T cells and the development of central memory T cells [50]–[54]. Therefore, to further investigate the effect of LAMP/*gag* immunization on the T cell response, we analyzed the phenotype and the expression of different cytokines by Gag-specific T cells (Figure 7). We observed that T cells obtained from mice immunized with LAMP/*gag* produced IFN-γ, IL-2 and TNF-α, as detected by intracellular cytokine staining (Figure 7A, B). TNF-α secretion was also evaluated by ELISA after T cell culture with either CD4- or CD8-restricted peptides and we confirmed that immunization with LAMP/*gag* induced an enhanced secretion of this cytokine by both T cell subpopulations, in comparison to immunization with native *gag* (Figure 7C).

Central memory T cells are characterized by the expression of chemokine receptors targeting to lymphoid organs. Therefore, we analyzed the expression of CCR7 in the CD4^+^ T cells obtained from native *gag* and LAMP/*gag* immunized mice and observed that LAMP/*gag* induced an increased expression of this receptor in CD4^+^ T cells (Figure 7D). Since naïve T cells also express this receptor we investigated whether the CCR7^+^ were primed cells by measuring IFN-γ production. Indeed, 5.15% of CD4^+^ T cells obtained from LAMP/*gag* immunized mice were CCR7^hi^IFN-γ^+^, in comparison to only 3.14% of the cells obtained from mice immunized with native *gag* (Figure 7E).

### CD8^+^ T cell activation induced by LAMP/*gag* immunization is dependent on CD4^+^ T cells

**Figure 8:**
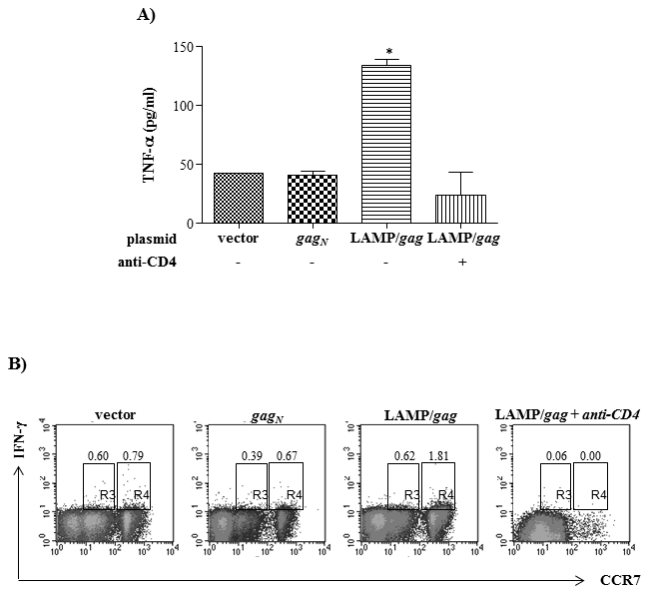
Enhanced immune response induced by LAMP/*gag* immunization is dependent on CD4^+^ T cells. Balb/c mice were treated or not with anti-CD4 antibody and immunized with LAMP/*gag*, as described in Material and Methods. Mice were also immunized with pITR vector or *gag_N_*, as controls. Fifteen days after the second immunization, total splenocytes were cultured with CD8-restricted gag peptide and cytokine production was analyzed. **A**) TNF-α secretion was analyzed by ELISA. **B**) The cells were incubated with PercP-anti-CD8, PE-anti-CCR7 and FITC-anti-IFN-γ and the expression of CCR7 and IFN-γ among CD8^+^ cells were analyzed by FACS. The data are representative of two independent experiments. * p<0,05.

In an effort to elucidate the importance of CD4^+^ T cell activation in our vaccination model we evaluated whether the activation markers detected in LAMP/*gag* immunized mice would also be elicited in the absence of CD4^+^ T cells. Mice were depleted of CD4^+^ T cells using specific anti-CD4 antibody and, then, immunized with LAMP/*gag*. After 2 DNA immunizations, total splenocytes were cultured with CD8-restricted Gag peptides and the secretion of TNF-α was evaluated. We observed that T cells obtained from animals immunized with LAMP/*gag* in the absence of CD4^+^ cells produced much lower amounts of TNF-α, comparable to the levels induced by immunization with native *gag* or pITR vector (Figure 8A). In addition, we observed a decrease in the expression of CCR7 in the CD4-depleted mice and a lower expression of IFN-γ among CCR7^hi^CD4^−^ or CCR7^l^°CD4^−^ cells (Figure 8B). The data demonstrated that CD4^+^ T cells were essential for the enhanced T cell response elicited by LAMP/*gag*.

## Discussion

Here, we dissected the roles of protein expression and cellular localization on the anti-HIV immune response. We have previously demonstrated that immunization of mice with a chimeric DNA plasmid containing HIV-1 p55*gag* inserted between luminal and transmembrane/cytoplasmic tail domains of LAMP-1 (LAMP/*gag*) elicited a much greater and prolonged Gag-specific immune response, when compared to immunization with native *gag* [28], [43], [44]. Given that increased protein expression/stability and differential antigen targeting can both influence the elicited immune response [21], [33], [55], we evaluated the mechanisms involved in these processes. We made several plasmid DNA constructs, in which the sequence of HIVp55*gag* was associated with intact or truncated domains of LAMP-1. The expression level and intracellular localization of the chimeric antigens were investigated in transfected cells and constructs inducing different patterns of expression and cellular targeting were inoculated in mice for analysis of the immune response.

We observed that the luminal domain was essential to LAMP-mediated increased expression of Gag, which was associated to an increased mRNA level in comparison to native *gag*. HIV structural proteins, including Gag, are poorly translocated to the cytoplasm [56]. The expression of Gag is hampered by the presence of inhibitory elements that are binding sites for cellular factors associated to mRNA instability, nuclear retention, and inefficient translation [57], [58]. During HIV infection, Gag expression requires the activity of the viral Rev protein, which is postulated to counteract the action of these factors [59]. Different strategies had been used to overcome *gag* poor translation in order to obtain an efficient protein expression [20]-[22], [60]. These strategies were supposed to either enhance translational efficiency, or to alter RNA export to the cytoplasm. The effect of LAMP on Gag expression, on the other hand, did not seem to be associated to protein stability or mRNA export to the cytoplasm. Our data suggests that addition of LAMP luminal domain upstream *gag* promoted enhanced increased levels of mRNA either by increased transcription or mRNA stability. Since no modification of the Gag inhibitory elements or codon usage was attempted, it is possible that the synthesis of LAMP/Gag chimera was being regulated by cellular mechanisms involved in the LAMP expression directed by signals present in the luminal domain.

The LAMP luminal domain also showed to be essential to Gag targeting to lysosomal compartments. Our previous studies showed that association of *gag_N_* with the transmembrane/cytoplasmic domain of LAMP was not sufficient to target the antigen to the MHCII-containing cellular compartments [43]. Therefore, we investigated the intracellular localization of the chimeras containing truncated sequences of the luminal domain. We observed that, different from native Gag, the truncated chimeras were present in cellular vesicles and were able to traffic to Golgi compartments. However, in spite of the presence of the LAMP targeting signal, they barely reached the lysosomal compartments, in contrast to the construct containing the whole luminal domain, which had been largely demonstrated to colocalize with endogenous LAMP [28], [43], [61].

The targeting of Gag mediated by the intact LAMP luminal domain also culminated in the secretion of the chimera partly associated to exosome-like vesicles. Several studies have associated antigen-containing exosomes to enhanced antigen presentation and activation of T cells. In fact, exosome-based cell free vaccines demonstrated to induce specific T cell responses *in vivo* [37]. Exosomes derived from antigen presenting cells (APC) contain MHC II and co-stimulatory molecules, and can directly stimulate CD4^+^ T cells [39], [40], [49]. Even when secreted by other cell types, the exosomes may transfer intracellular antigens directly to APCs and promote antigen cross-presentation [42]. This is particularly interesting in the context of a DNA vaccine. Although inoculation of DNA plasmids can induce immune response after direct transfection of APCs, most of the inoculated DNA is probably captured by other cell types. In this case, antigens that remain cell-associated may not be efficiently delivered to APCs and need to be secreted or transferred to APC by cross-priming to induce immune response [62], [63]. Indeed, association of tumor-derived antigens, HIV-gp120, and other antigens with signal delivering to exosomes was shown to increase T and B cell responses [37], [38], [64], [65].

We could not detect native Gag in the supernatant of transfected cells, in spite of its well-known ability to generate secreted virus-like particles (VLP). It is possible that the amount of synthesized protein in its native form, in the absence of Rev, was not sufficient to allow VLP generation and release. Secreted HIV Gag VLPs usually bud from the plasma membrane and not from vesicles originated from endolysosomes [17]. Accordingly, LAMP_T2-lum_/Gag chimera, which showed to be strongly expressed, was secreted by the transfected cells, although not significantly detected in the exosome fraction. These data suggest that the targeting of Gag to endolysosomal vesicle by the whole luminal domain of LAMP influenced its secretion pathway.

Given that we constructed plasmids that differ in their expression level and cellular traffic, we were able to evaluate the relative importance of these features for the Gag-specific immune response elicited by LAMP/*gag* DNA vaccines. Mice were immunized with (i) native *gag*, (ii) the truncated LAMP/*gag* chimeras (LAMP_T1-lum_/*gag* or LAMP_T2-lum_/*gag*), and (iii) the intact LAMP/*gag,* and the T and B cell responses were analyzed. It was observed that the anti-HIV antibody response elicited by these plasmids was proportional to the length of the luminal domain. Immunization with the truncated LAMP_T2-lum_/*gag* plasmid, which induced the same expression level as the intact LAMP/*gag*, elicited a similar level of anti-HIV serum IgG antibodies, indicating that protein expression level was proportional to the magnitude of acute antibody response. Analysis of the induced T cell response, however, demonstrated that, although the level of activation was also proportional to Gag expression, all the truncated plasmids elicited a significantly lower IFN-γ production by CD4^+^ or CD8^+^ T cells than the plasmid containing the whole LAMP luminal domain.

Gag targeting to the endolysosomal compartments may not only facilitate its presentation by MHC II molecules and increase CD4^+^ T cells activation, but also, the secretion of Gag may improve cross-priming and directly activation CD8^+^ T cells. It has been described that dendritic cells pulsed with a particulate form of the hepatitis B antigen processed the antigen in the endolysosomal compartment and efficiently primed CTL, inducing a higher T cell response in comparison to equimolar concentration of the peptide in a non-particulate form [66]. Although there is no experimental evidence demonstrating Gag secretion *in vivo*, the fact that the truncated LAMP_T2-lum_/*gag* plasmid did not induce the same level of T cell response, in spite of a high level of Gag expression, indicates that Gag traffic may be the main event regulating the potent T cell activation mediated by LAMP/*gag*. Accordingly, previous studies demonstrated that DNA vaccines that generate Gag secreted as VLP, or in a soluble form, induce different levels of T and B cell activation, which were also different from the response induced by cytoplasmic Gag [33].

Since T cell activation is essential to induce memory response, we evaluated whether the level of antigen-specific IgG antibodies induced by LAMP_T2-lum_/*gag* would be sustained in spite of the low T cell activation induced. Analyses of the IgG antibody titer at different time points after immunization demonstrated that the anti-HIV IgG levels induced by LAMP_T2-lum_/*gag* rapidly decreased, in contrast to the levels induced by intact LAMP/*gag*, which was maintained for at least three months after vaccination. These data demonstrated that the T cell activation associated to differential antigen traffic was essential to promote prolonged antibody response after immunization.

CD4^+^ T cells have also been reported to be essential for the activation of CD8^+^ T cells. We demonstrated here that LAMP/*gag* immunization induced polyfunctional CD4^+^ T cells able to produce IFN-γ, TNF-α and IL-2. Furthermore, those cells presented a phenotype of central memory T cells, expressing CCR7 and IFN-γ. The importance of CD4^+^ T cells for the whole T cell activation was clearly evidenced after immunization of mice depleted of CD4^+^ T cells, where we observed almost total abrogation of the TNF-α and IFN-γ production by CD8^+^ T cells.

Finally, we demonstrated that the association of Gag with LAMP luminal domain, in the absence of transmembrane/cytoplasmic domain, was sufficient to modulate Gag traffic and anti-Gag immune response. Therefore, the traffic signals present in this region seemed to be the main event regulating the immune response.

In summary, we described the mechanisms involved in the immune response induced by LAMP/*gag* chimeric DNA and demonstrated that the luminal domain of LAMP is a key element in this construct, inducing higher Gag expression, and its traffic through the endolysosomal and secretory pathways. Analysis of the immune response elicited by chimeric DNA plasmids containing truncated sequences of LAMP-1 suggested that increased protein expression was sufficient to induce an enhanced, but transitory antibody response; however, increased T cell response depended on antigen targeting. These findings further enhance our knowledge regarding LAMP-mediated enhanced immunity and may contribute not only for the development of novel anti-HIV vaccines, but also to general vaccinology field.

## Supporting Information

Figure S1 **Association of p55** ***gag*** **with LAMP luminal domain is sufficient to induce a T cell immune response.** **A-B**) Balb/c mice were immunized twice with the indicated plasmids, and fifteen days later, total splenocytes were cultured with p55Gag protein and IFN-γ secretion was analyzed by ELISA (**A**); or the cells were cultured with MHC I restricted Gag epitope AMQMLKETI65-73 and the expression of IFN-γ among CD8^+^ cells were evaluated by FACS (**B**). The data are representative of three independent experiments. * p<0,05.
(TIF)
