# Supplementary figures and images for: Regulation of HIV-Gag Expression and Targeting to the Endolysosomal/Secretory Pathway by the Luminal Domain of Lysosomal-Associated Membrane Protein (LAMP-1) Enhance Gag-Specific Immune Response
Source: PLoS One. 2014 Jun 16;9(6):e99887. doi: 10.1371/journal.pone.0099887 (PMC4059647; doi:10.1371/journal.pone.0099887)

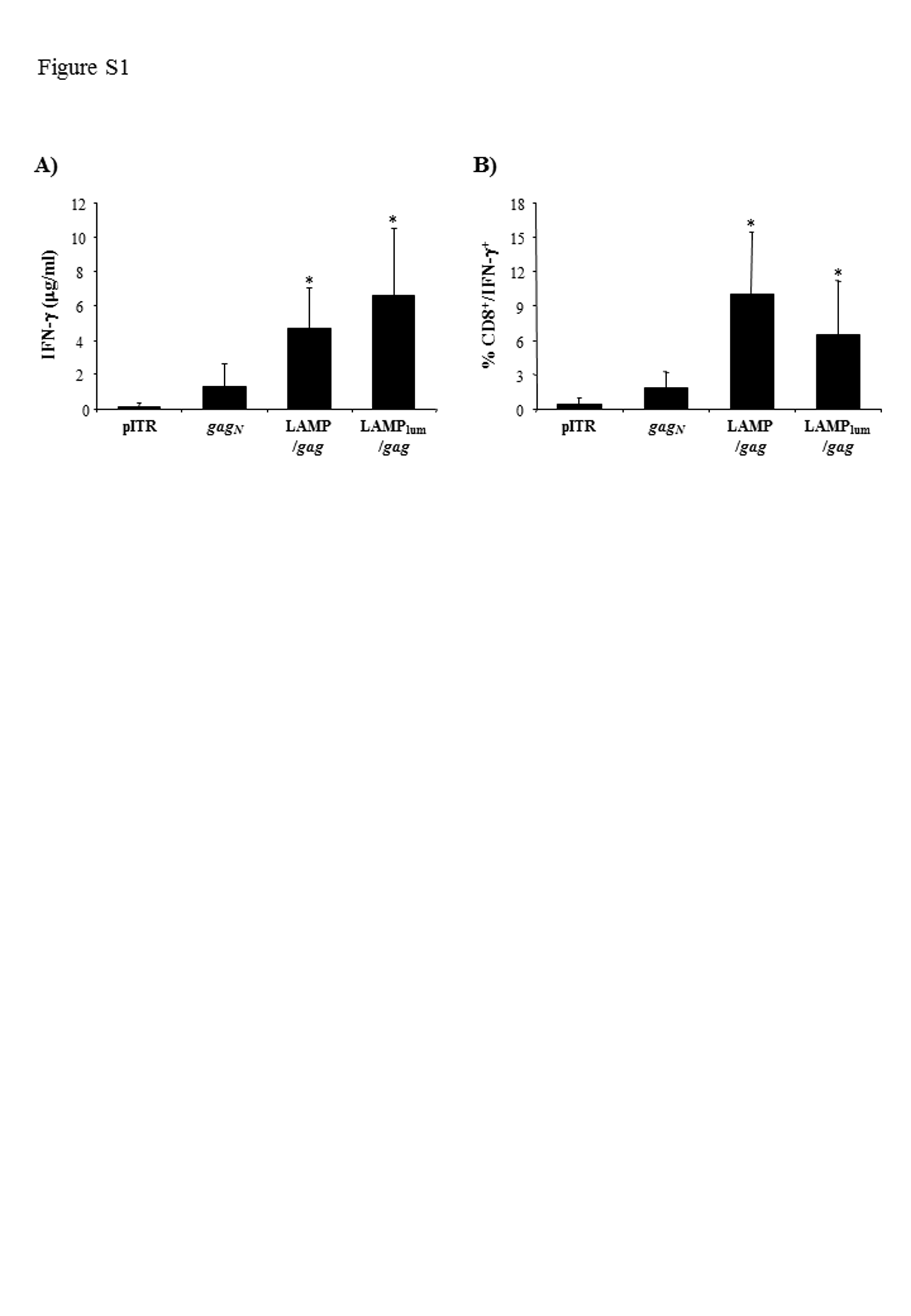

Supplement: Figure S1 — Association of p55 gag with LAMP luminal domain is sufficient to induce a T cell immune response. A-B) Balb/c mice were immunized twice with the indicated plasmids, and fifteen days later, total splenocytes were cultured with p55Gag protein and IFN-γ secretion was analyzed by ELISA (A); or the cells were cultured with MHC I restricted Gag epitope AMQMLKETI65-73 and the expression of IFN-γ among CD8+ cells were evaluated by FACS (B). The data are representative of three independent experiments. * p<0,05. (TIF) [file pone.0099887.s001.tif]
